# Smad-dependent mechanisms of inflammatory bone destruction

**DOI:** 10.1186/s13075-016-1187-7

**Published:** 2016-12-01

**Authors:** Michelle Fennen, Thomas Pap, Berno Dankbar

**Affiliations:** Institute of Experimental Musculoskeletal Medicine, Westfalian Wilhelms-University Münster, Münster, Germany

**Keywords:** Rheumatoid arthritis, Osteoclastogenesis, Smad, TGF-β1, Myostatin, Activin A

## Abstract

Homeostatic bone remodelling becomes disturbed in a variety of pathologic conditions that affect the skeleton, including inflammatory diseases. Rheumatoid arthritis is the prototype of an inflammatory arthritis characterised by chronic inflammation, progressive cartilage destruction and focal bone erosions and is a prime example for a disease with disturbed bone homeostasis. The inflammatory milieu favours the recruitment and activation of osteoclasts, which have been found to be the cells that are primarily responsible for bone erosions in many animal models of inflammatory arthritis. Among the inflammatory modulators, members of the transforming growth factor (TGF)-β super family are shown to be important regulators in osteoclastogenesis with Smad-mediated signalling being crucial for inducing osteoclast differentiation. These findings have opened a new field for exploring mechanisms of osteoclast differentiation under inflammatory conditions. Recent studies have shown that the TGF-β superfamily members TGF-β1, myostatin and activin A directly regulate osteoclast differentiation through mechanisms that depend on the RANKL–RANK interplay. These growth factors transduce their signals through type I and II receptor serine/threonine kinases, thereby activating the Smad pathway. In this review, we describe the impact of inflammation-induced Smad signalling in osteoclast development and subsequently bone erosion in rheumatoid arthritis.

## Background

Sustained bone remodelling is important for healthy conservation of mobility and structure of the skeleton, in which bone matrix is constantly resorbed by osteoclasts and subsequently replaced with new bone by osteoblasts. Thus, bone remodelling requires the coordinated actions of the bone-resorbing osteoclasts and the bone-forming osteoblasts [1, 2].

An imbalance of this homeostatic process through a perturbation by inflammatory cytokines, growth factors and hormones can result in skeletal abnormalities, such as osteoporosis, osteopetrosis or rheumatoid arthritis (RA) [3, 4]. In RA, a variety of pathologic conditions affect the skeleton, in which altered levels of proinflammatory cytokines stimulate bone resorption in the inflamed joints.

RA is a systemic disorder characterised by chronic inflammation, hyperplasia of synovial lining cells and the deep infiltration of the inflamed and hyperplastic synovium into the joint structures. Pathology starts with a painful inflammation of the synovial tissues, tendon sheaths and bursae and eventually results in the progressive destruction of the articular cartilage and subchondral bone of the affected joints [5, 6]. Focal bone erosions occur at the interface between cortical bone and immigrant pannus tissue and at the subchondral bone where the pannus invades the bone marrow [7]. Arthritic bone erosions are mediated by osteoclast precursors and mature osteoclasts [8].

Besides bone erosion, extensive inflammation is present during the progression of RA and inflammatory mediators released by cells of the invasive pannus are involved in the inflammatory process in RA, resulting in destruction of physiological tissue barriers. Many of the cytokines and growth factors implicated in the inflammatory process have also been demonstrated to impact directly or indirectly on osteoblast and/or osteoclast differentiation and function [9, 10]. Here, we want to focus on several members of the transforming growth factor (TGF)-β super family that influence osteoclast development and bone destruction in arthritis.

## RANKL-mediated osteoclast development and activation

Cells of the monocyte/macrophage lineage, which are located in the synovial tissue of inflamed joints in RA, serve as potential osteoclast precursors and drive osteoclast formation by specific molecular signal induction [11, 12]. Osteoclasts differentiate from these progenitors in the presence of macrophage colony stimulating factor (M-CSF; also called CSF-1) and receptor activator of nuclear factor κB ligand (RANKL). Osteoblasts secrete the cytokines M-CSF as well as RANKL, which is also expressed by T lymphocytes, osteoclasts and fibroblast-like synovial cells as part of the pannus [11, 13]. M-CSF binds to its receptor FMS on the surface of premature osteoclasts and activates signalling pathways essential for proliferation and survival of osteoclast precursors [12].

Subsequent binding of soluble or membrane bound RANKL to the receptor activator of nuclear factor κB (RANK) on the cell surface of osteoclast precursors and mature osteoclasts leads to the formation, activation and survival of mature osteoclasts. The action of RANKL is regulated by osteoprotegerin (OPG), which is secreted by osteoblasts and impairs the binding of RANKL on its specific receptor RANK by binding of soluble RANKL as a decoy receptor [14–17].

Binding of RANKL to RANK leads to the recruitment of the adaptor protein TNF receptor-associated factor 6 (TRAF6), which results in a trimeric complex formation of TRAF6, TAB2, TAB1 and TGF-beta-activated kinase 1 (TAK1) [18, 19]. TAK1 is activated, which subsequently leads to the activation of downstream signalling pathways such as NF-κB, p38 mitogen-activated protein kinase (p38 MAPK) and c-Jun N-terminal kinase 1 (JNK1) [12, 18, 20] and the induction of c-fos/c-jun hetero- and homodimeric transcription factors (AP-1) [12].

However, the master transcription factor of osteoclast differentiation is nuclear factor of activated T-cells cytoplasmic 1 (NFATc1), which is stimulated by RANKL via TRAF6, c-Fos and NF-κB signalling as well as through the activation of immunoreceptor tyrosine-based activation motif containing receptors (ITAMs), e.g. FcγR and recruitment of spleen tyrosine kinase (SYK) by the activation of phospholipase Cγ (PLCγ). Interestingly, NFATc1 can auto-regulate its expression through binding on its own promoter through the NFAT-binding side [21]. Activation and nuclear translocation of NFATc1 is mediated by calcineurin upon RANKL-induced Ca^2+^ oscillations [12, 18, 20, 22]. Following translocation into the nucleus, NFATc1 together with other transcription factors like AP-1, microphthalmia-associated transcription factor (MITF), PU.1 or CREB induce the expression of various osteoclast-specific genes, such as those encoding cathepsin K, tartrate-resistant acid phosphatase (*TRAP*), calcitonin receptor, osteoclast-associated receptor (*OSCAR*) and β_3_-integrin [21]. Finally, binding of TRAF6 potentiates the activation of c-Src/SFK kinase, leading to an activation of phosphatidylinositol 3 kinase (PI3K), which, in turn, leads to an activation of protein kinase B (PKB), which has an anti-apoptotic effect on osteoclasts by inactivation of pro-apoptotic proteins such as caspase-9 [18, 23].

The activation of mature osteoclasts by RANKL involves the expression of genes encoding TRAP, calcitonin receptor (CTR), H^+^-ATPase, cathepsin K, matrix metalloproteinase 13 (MMP13), MMP9, tumour necrosis factor (TNF)α, interleukin (IL)-6, IL-1 or carbonic anhydrase 2 (CA2) [12]. Transcription of these genes results in an increased bone resorption via the attachment of mature resorptive osteoclasts to the surface of the bone. Polarization of the osteoclast cytoskeleton, formation of the “ruffled border” and integrin α_V_β_3_-mediated binding of the osteoclast to the bone surface, formation of an acidic microenvironment by the osteoclast and secretion of enzymes like cathepsin K, TRAP, H^+^-ATPase or MMP13 facilitate dissolving of bone mineral and degradation of bone matrix [12].

**Fig. 1:**
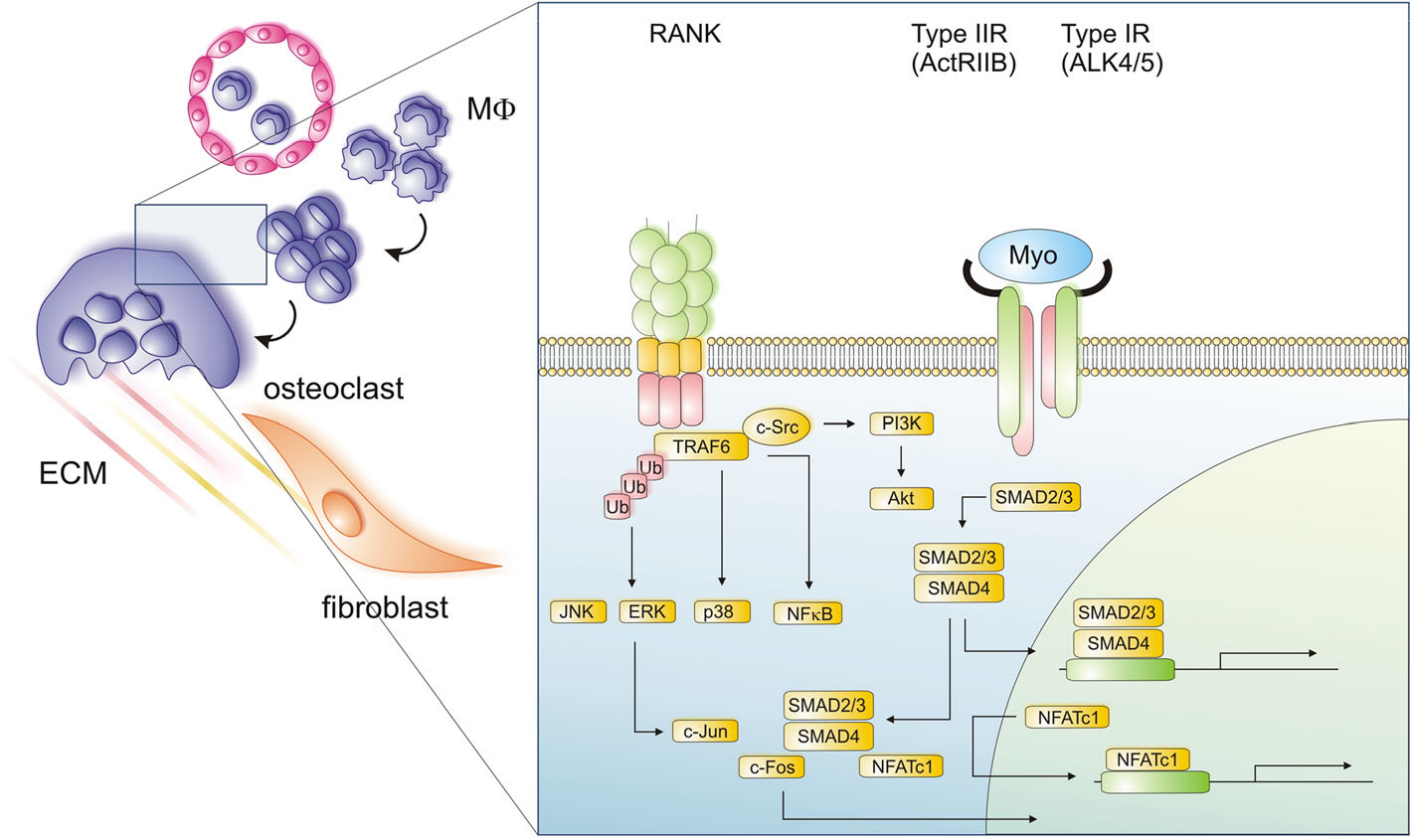
Osteoclasts arise from hematopoietic monocyte/macrophage precursors upon interaction of RANKL with its receptor RANK. Both are key regulators of bone remodelling and essential for the development, activation and survival of osteoclasts. Binding of RANKL to RANK leads to the recruitment of TRAF6, which activates Akt (survival), NF-κB and the mitogen-activated protein kinases p38, ERK and JNK, resulting in the induction of the transcription factors c-Jun, c-Fos and NFATc1, all crucial for osteoclast differentiation. As an important signalling cascade promoting the RANKL-induced osteoclastogenesis, the Smad signalling pathway, which can be activated by transforming growth factor (TGF)-β family members, including TGF-β, activin and myostatin, has been identified. By way of example, myostatin signals through the ActRIIB-ALK4/5 heterodimer to activate Smad2/3, which subsequently translocates directly to the nuclear compartment or binds first to NFATc1 and then translocates into the nucleus, both leading to enhanced expression of several osteoclast-specific target genes, including NFATc1 itself. Smad-mediated translocation of c-Fos, which has been demonstrated upon TGF-β stimulation during RANKL-mediated osteoclastogenesis is also indicated in the figure. *ECM* extracellular matrix, *Ub* ubiquitin

During the progression of RA, the inflammatory milieu of affected joints favours the recruitment and activation of osteoclasts, leading to a progressive destruction of joint structures. Among the known inflammatory modulators, members of the TGF-β super family have been shown to be important regulators of osteoclastogenesis with Smad-mediated signalling being crucial for inducing osteoclast differentiation. These findings have opened a new field for exploring inflammatory mechanisms of osteoclast differentiation. Recent studies have shown that the TGF-β super family members TGF-β1, myostatin and activin A directly regulate osteoclast differentiation through a mechanism dependent on RANKL–RANK interplay (Fig. 1).

## The TGF-β family and their signalling

The TGF-β superfamily is divided into two functional groups, the TGF-β-like group and the bone morphogenetic protein (BMP)-like group. The TGF-β-like group consists of all TGF-βs, activins, nodals and a few growth and differentiation factors (GDFs), whereas the BMP-like group involves BMPs, most GDFs and the anti-Müllerian hormone (AMH) [24]. These structurally related and secreted polypeptides are encoded by 33 genes and characterised by a signal peptide, a large pro-domain and a C-terminal mature polypeptide including seven or nine cysteine residues. These mature polypeptides form disulphide-bonded dimers that are involved in a high variety of biological activities such as tissue development and physiology [25].

Only seven type I (also termed activin receptor-like kinases (ALKs)) and five type II receptors are encoded by the mammalian genome and serve as a receptor repertoire for diverse TGF-β-like and BMP-like proteins. TGF-β and activin proteins bind to the tetrameric cell surface complex consisting of two transmembrane kinase pairs, type II receptor kinase (TβRII and ActR-II/ActR-IIB, respectively) and type I receptor kinase (TβRI/Alk-4 and ActR-I/Alk-2 or ActR-IB/Alk-4, respectively). The receptor complex is stabilised upon ligand binding and intracellular signals mediated by Smad proteins are induced by distinct combinations of type I and type II receptors depending on the bound ligand [26, 27].

In mammals, eight Smad proteins are known, which are divided into three subclasses: the receptor-regulated Smads (R-Smad), the common-mediator Smads (Co-Smads) and the inhibitory Smads (I-Smads) [26, 28]. It is well known that TGF-β-like proteins induce their intracellular signals via phosphorylation of R-Smad2/3 by the type I receptor kinase while BMPs phosphorylate and activate R-Smad1/5/8 [27, 29]. Co-Smad-4 can bind to phosphorylated R-Smad2/3 and the trimeric complex is then translocated into the nucleus where it binds to high-affinity DNA-binding transcription factors and conducive Smad-binding DNA sequences at regulatory promoter sequences to trigger transcription responses on target genes [25, 26]. I-Smad7 is an inhibitory regulator of the TGF-β/BMP signalling pathway which blocks signal transduction upon stably interacting with the TGF-β-, activin- or BMP-activated type I receptors [26, 30]. Thereby, TGF-β promotes the transcription of I-Smad7 as well as promotes the degradation of I-Smad7 by activation of the Smad3-dependent Smurfs/arcadia-mediated ubiquitin-proteasome degradation pathway [30–32].

## Modulation of osteoclastogenesis by TGF-β1

TGF-β1 is a factor with multiple biological properties, e.g. cell differentiation, proliferation, migration, apoptosis, autophagy or production of extracellular matrix [32–34]. It is expressed in the perichondrium and in chondrocytes of mouse embryonic cartilage, in the periosteum and in osteocytes in mouse embryonic bones and by bone marrow cells, chondrocytes, osteoclasts and in the cartilaginous matrix in adult mice [33, 35, 36]. It is secreted in a latent form and stored in the extracellular matrix. Interestingly, opposing effects of TGF-β1 have been described and its function on osteoclastogenesis and bone resorption is controversial On the one hand, TGF-β1 has the ability to increase OPG secretion by osteoblasts, leading to an inhibition of osteoclast maturation in coculture [14–17]. On the other hand, TGF-β1 can facilitate bone resorption by recruitment of osteoclast precursors to the bone environment and promoting osteoclast differentiation in the presence of RANKL, which can be completely blocked by addition of OPG in vitro [16, 33, 37]. Moreover, TGF-β1 can act dose-dependently on osteoclast differentiation (reviewed by Janssens et al. [33]) and stimulate osteoclast development through enhanced expression of RANK on the surface of monocytic/pre-osteoclastic cells [38].

During osteoclast differentiation, TGF-β binds to its type II receptor to recruit and phosphorylate type I receptor, leading to the phosphorylation of R-Smad2 and 3. Activated p-R-Smad2/3 generates an active Smad complex by binding to Co-Smad4, which translocates into the nucleus and induces the transcription of several target genes. In this context, the cooperation of the activated Smad complex with the co-transcription factor c-Fos is necessary for nuclear translocation and subsequent DNA binding [39]. In detail, TGF-β enhances the RANKL-mediated translocation of the activated Smad complex (p-R-Smad2/3 and p-c-Fos) into the nucleus, followed by c-Fos-mediated binding of the activated complex to the *Nfatc1* gene. This then drives the expression of NFATc1, which is an essential factor in the regulation of osteoclast differentiation [39]. Furthermore, TRAF6 binds to Smad3 via its MH2 domain, which is important for the RANKL/RANK signal transduction [20]. Importantly, inhibition of TGF-β impairs the formation of the RANKL-induced TRAF6 complex, resulting in impaired intracellular signal transduction through p38 MAPK, JNK or NF-kB signalling pathways. Finally, TGF-β1 mediates osteoclast survival by increased ALK5/TβR1 expression and activation of the R-Smad2/3 complex, which then activates the pro-survival factor Mcl-1 in mature osteoclasts [40].

## The role of TGF-β1 in inflammatory bone destruction

TGF-β1 is expressed by cells of the synovial lining layer and macrophages as well as in the vascular endothelium of synovial tissues of RA patients compared to osteoarthritis (OA) and healthy controls [41], and synovial fluid of RA patients contains TGF-β1, which indicates that TGF-β1 may be involved in the destruction of the affected joints [16, 42]. Following this, the role of TGF-β1 was precisely examined in mouse models of RA but conflicting results were described for TGF-β1 in murine models of arthritis [34].

Mice with collagen-induced arthritis (CIA) show a reduced occurrence and severity of arthritis when TGF-β1 is intraperitoneally injected at late stages of disease progression [34, 43] and TGF-β1-transduced mouse bone marrow-derived mesenchymal stem cells, intraperitoneally injected in CIA mice, move to the affected joints and lead to reduced osteoclastogenesis verified by decreased expression of TRAP, cathepsin K or NFATc1 as well as decreased destruction of the articular cartilage, synovial hyperplasia, bone erosion and infiltration by inflammatory cells [44]. Accordingly, treatment of CIA mice with an anti-TGF-β1 antibody leads to increased disease progression [45]. However, others recently showed that inhibition of TGF-β1 with the specific TGF-β blocking peptide p17 has only a minor or no effect on clinical severity, joint inflammation and cartilage destruction in the CIA model [46]. In contrast, early studies showed that the injection of TGF-β1 in healthy knee joints of mice and rats induced joint inflammation, with neutrophil recruitment, synovial inflammation and immune cell infiltration [34, 47]. In a model of chronic erosive polyarthritis, anti-TGF-β1 and -2 treatment led to reduced inflammatory cell accumulation and decreased tissue degradation and swelling [48].

However, TGF-β1 combined with other cytokines seems to play a pivotal role in the progression of RA, although rather indirectly in osteoclast-mediated bone destruction. In synovial fibroblasts of RA patients, TGF-β1 induces the activity of the transcription factors NF-κB and AP-1, which are involved in the development of inflammation, and enhances the expression of inflammatory mediators such as TNFα, IL-1β, IL-6 and IL-8, degrading enzymes such as MMP1 and MMP3 as well as the angiogenic factor VEGF [49, 50]. Additionally, TGF-β1 has a synergistic effect with TNFα on the expression of IL-1β in RA synovial fibroblasts [49] and induces the proliferation of synovial fibroblasts through platelet-derived growth factor (PDGF)-AA and cMyc induction, resulting in a massive synovial hypertrophy in the affected joints [50].

Moreover, inhibition of the TGF-β1 type I receptor kinase has a preventive effect on the development of arthritis in mice [50] and TGF-β1 induces the expression of transmembrane receptor protein tyrosine phosphatase κ (RPTPκ), which seems to be involved in cytoskeletal reorganisation, migration and invasiveness of RA synovial fibroblasts, promoting fibroblast migration through dephosphorylation of the tyrosine-protein kinase v-src avian sarcoma viral oncogene homolog (SRC) [51].

Finally, TGF-β1 promotes the differentiation of T_H_17cells, which are recognised as one of the key players inducing osteoclastogenesis via IL-17A under inflammatory conditions such as RA [52–56].

T_H_17 cells differentiate from naïve CD4+ T cells in the presence of TGF-β1, IL-6 and IL-21 in mice, whereas in humans IL-6 and IL-1β but not TGF-β1 are essential. These cytokines are involved in the expression of the transcription factor RORγt [57–61], which is then required for de novo T_H_17 cell differentiation [62–64] from naïve CD4+ T cells through the upregulation of IL-7Rα and downregulation of CD27 and IL-2 [65]. IL-23, a dendritic cell-derived cytokine, is sufficient for IL-17 release and stabilization of T_H_17 cells and therefore essential for their pathogenicity in vivo [66, 67], but early differentiation occurs independently of IL-23. If IL-23 is missing, T_H_17cells are not terminally differentiated and will go into apoptosis. Normally, IL-23 promotes the establishment of a large number of T_H_17 cells in the lymph nodes, which then exit the lymph node and migrates to the inflamed tissue, which is also supported by the local expression of IL-23 [65]. The differentiated T_H_17 cells express IL-17A, IL-17 F, IL-21 and IL-22 [68] and these cells are able to produce RANKL and TNFα, which are important factors in RA [21, 61, 69, 70]. In the synovial fluid of patients with RA, levels of IL-17A are significantly increased in comparison to patients with OA [71]. IL-17A indirectly leads to the expression of RANKL by fibroblast-like synoviocytes (FLS) and osteoblasts, subsequently leading to the differentiation of osteoclast precursors into mature osteoclasts [21, 60, 61, 69–71]. Additionally, IL-17A induces the expression of TNFα, IL-6 and IL-1β by synovial macrophages, which indirectly leads to the expression of RANKL by osteoblasts [61, 72], which in turn promotes osteoclast differentiation as well [21, 55, 56, 64–66]. Furthermore, the production of IL-6, IL-8, MMP-3 as well as granulocyte colony-stimulating factor by human FLS is additively/synergistically induced by IL-17A in combination with TNFα [73] and the inhibition of IL-17 in hTNFtg mice leads to delayed bone erosion and cartilage destruction, whereas inflammation remains unaffected [73, 74].

In summary, TGF-β1 appears to play a pivotal role in the progression of RA but rather indirectly in combination with other factors involved in osteoclast-mediated bone destruction.

## Modulation of osteoclastogenesis by myostatin

Myostatin (also known as GDF-8), another member of the highly conserved TGF-β family, is expressed mainly in skeletal muscle and acts as an autocrine/paracrine inhibitor of skeletal muscle growth [75]. In accordance with this, deletion of the myostatin gene or a loss of function mutation leads to muscle hypertrophy and hyperplasia with an approximate doubling of muscle mass in mice as well as in other mammals such as dogs, sheep, cattle or humans [75–78].

However, several findings indicate that myostatin also plays a role in regulating bone development and that inhibition of myostatin diminishes bone resorption and improves bone formation. Indeed, treatment of mice with an anti-myostatin decoy receptor (ActRIIB-Fc) results in a significant increase in bone mass [79] and myostatin-deficiency leads to increased callus formation and bone volume in the ossified callus after osteotomy [80]. Of note, myostatin is expressed during the inflammatory phase of fracture healing [81] and it can be suggested, therefore, that the early expression of myostatin in the callus leads to suppressed recruitment and proliferation of progenitor cells at the place of fractured bone [80].

Myostatin is secreted and the latent form of myostatin, dimerised by disulphide bonds at the C-terminal region, circulates in the blood. Upon cleavage by the BMP1/Tolloid matrix metalloproteinase, the propeptide is released from the C-terminal domain of myostatin but the active protein can still be inhibited by its propeptide and other endogenous inhibitors such as follistatin or follistatin-related gene (FLRG) [82, 83].

Typically, myostatin transduces intracellular signals by binding to the heterodimeric receptor complex, composed of the activin type II receptor 2B (ACVR2B) and the type I receptor ALK4 (known as ACVR1B) or ALK5 (TβR-I), through multiple intracellular signalling cascades, including the SMAD and MAPK pathways [84]. By binding of myostatin to the heterotetrameric receptor complex, the dormant type I receptor kinase ALK4 and/or ALK5 is activated, which leads to a subsequent activation of R-Smad2 and R-Smad3. Co-Smad4 binds to the activated complex and the whole complex is translocated into the nucleus, where it activates the transcription of several genes. The myostatin-induced signalling cascade is regulated through a negative feedback loop via I-Smad7, in which I-Smad7 inhibits the myostatin promotor activity and myostatin transcription via cooperation with Smurf1 [83, 85].

## The role of myostatin in inflammatory bone destruction

A recent study demonstrated that myostatin is significantly involved in inflammatory bone loss in RA by directly promoting osteoclast formation. Myostatin is expressed in synovial tisssues of RA patients in contrast to those of OA patients, indicating that myostatin is upregulated in synovial-like fibroblasts during the inflammatory progression of RA in humans. Additionally, myostatin is expressed in synovial membranes of human TNFa transgenic (hTNFtg) compared to wild-type mice [86]. These mice spontaneously develop a TNFα-dependent chronic destructive arthritis. Overexpression of TNFα in mice is sufficient to trigger full-blown destructive arthritis with synovial inflammation, cartilage damage and bone destruction [87].

Moreover, the lack or pharmacological inhibition of myostatin in hTNFtg mice led to reduced severity of RA and decreased bone erosion in the joints associated with a decreased number of mature osteoclasts as well as a significantly diminished inflammatory response [86].

Interestingly, myostatin by itself is not able to induce the formation of mature osteoclasts from bone marrow-derived macrophages (BMMs), but is able to enhance the ability of osteoclast precursors to differentiate into mature osteoclasts in a RANKL/M-CSF-dependent manner. The treatment of BMMs with myostatin, M-CSF and RANKL leads to the establishment of very large osteoclasts with a huge cytoplasmic compartment and a large number of nuclei in comparison to mature osteoclasts cultivated under normal conditions (M-CSF, RANKL). Besides a paracrine role, myostatin has an additionally autocrine role as demonstrated by decreased RANKL-induced osteoclastogenesis by myostatin-deficient cells and lower osteoclast numbers in the tibea of myostatin-deficient mice [86].

During osteoclastogenesis, myostatin binds to its heterodimeric receptor complex on the surface of precursor and mature osteoclasts and subsequent activation of ALK4 or ALK5 leads to the recruitment of R-Smad2. R-Smad2 is activated via phosphorylation (p-R-Smad2) and binds to NFATc1. These complexes are then translocated into the nucleus of precursor osteoclasts to enhance expression of several osteoclast-specific target genes. In detail, myostatin enhances the RANKL-dependent expression of integrin αvβ3, DC-STAMP and calcitonin receptor as well as NFATc1 itself. However, myostatin has no effect on the activation of the MAPKs p38α, c-Jun N-terminal kinase (JNK) and the extracellular signal-regulated kinase 1/2 (ERK1/2) nor the NF-κB pathway during osteoclast differentiation. However, apart from a decrease in osteoclast development and bone destruction, a strong reduction in inflammation can be observed in myostatin-deficient arthritic mice [86]. This in vivo observation is of great importance since under inflammatory conditions there is local and/or systemic alteration in the levels of proinflammatory cytokines and growth factors that are known to stimulate bone resorption in vitro and in vivo [9, 10]. Thus, beside a direct effect on osteoclast development, myostatin appears to additionally modulate joint inflammation.

## Modulation of osteoclastogenesis by activin A

Activin A, a homodimer, is structurally composed of two βA subunits (βAβA) connected with disulphide bonds. Activin A acts as an autocrine as well as paracrine regulator and is expressed in a number of tissues such as ovary, pituitary gland, brain, placenta and bone marrow. In the bone marrow, activin A is most likely expressed by bone marrow macrophages, monocytes, osteoblasts, osteoclasts and endothelial cells [88–91].

Activin A acts as a powerful co-factor of the M-CSF/RANKL-induced differentiation of pre-osteoclasts and inhibition of activin A by its soluble receptor can abrogate osteoclast differentiation, indicating that activin A might be an essential factor for osteoclast differentiation [89, 92, 93]. Interestingly, activin A also stimulates osteoblast differentiation from murine bone marrow and promotes fracture healing in a rodent facture model, suggesting that activin A is involved in both bone formation and bone resorption during bone remodelling [94, 95]. Indeed, inbibition of activin A suppressed osteoclastic bone resorption, e.g. bone lesions and metastasis, and stimulated osteoblastic bone formation in mouse models of myeloma and breast cancer, respectively [96, 97]. In contrast, it has also been shown that activin A inhibits the differentiation of osteoblast precursors of rat calvaria [88]. Most interestingly, autocrine activin A affects the late phase of osteoblast differentiation, in which activin inhibits bone matrix formation and mineralisation, indicating that activin A might control bone quality during remodelling as well as pathological mineralisation [98].

Similar to myostatin, activin A signals through a combination of ACVR2B or ACVR2A and ALK4/7 receptors, thereby activating the Smad, NF-κB, ERK and p38 signalling pathway [92, 99]. Activin A has a stimulatory effect on RANK-mediated osteoclastogenesis via the activation of the IkBα/NF-κB pathway. This in turn induces the transcription of RANK by osteoclast precursor cells, leading to a higher susceptibility to RANKL [93]. In contrast to activin A, myostatin has the ability to activate R-Smad-2 but did not activate MAPK or NF-κB in osteoclasts, indicating that, despite using common receptors, they can activate different pathways [86, 88].

Recently, a conflicting report on osteoclast formation by activin A has been published suggesting that activin A acts as a local regulator during osteoclast development at multiple stages of osteoclast maturation by having a dominant negative effect on RANKL-stimulated osteoclast motility as well as osteoclast lifespan [100]. Interestingly, by testing whole bone marrow cultures or enriched osteoclast progenitor cultures they found enhanced RANKL-mediated osteoclast development similar to what has been described previously [89, 92, 93]. However, activin A had no effect on stroma-free BMMs stimulated with M-CSF and RANKL, suggesting that the stimulatory effect of activin A on osteoclastogenesis is mediated by other non-macrophage like cells in the bone marrow. From their study, they claim, that activin A has no pro-osteoclastogenic effect on stroma-free BMMs, whereas activin A acts as a negative regulator of RANKL-induced osteoclast motility by activation of R-Smad2 and Akt1, which leads to activation of IκBα, a negative regulator of the NF-κB pathway [100].

In contrast to the inhibition of osteoblastic bone formation in myeloma and breast cancer mouse models by activin A [96, 97], it has been demonstrated very recently that, in fibrodysplasia ossificans progressive (FOP), a constitutively activating mutation (R206H) of the bone morphogenetic protein type 1 receptor activin-like kinase 2 (ACVR1/ALK2) leads to an extensive ossification of skeletal muscle, fascia, ligaments and tendons. Due to this single nucleotide mutation at the position 617G-A, activin A could activate the mutated type I receptor ACVR1/AlK2, which normally can only be activated by BMPs [101–103]. During FOP progression, the differentiation of ACVR1(R206H)-expressing cells towards the osteoblast linage is enhanced through BMP-induced hetero-oligomeric type-II/ACVR1(R206H) complex hyperactivation [103]. Hatsell et al. [101] as well as Hino et al. [102] demonstrated that activin A binds to the mutated ACVR1(R206H) receptor and induces the phosphorylation of SMAD 1/5/8. Interestingly, activin A normally binds to ACVR1/ALK-2, acting as an antagonist by blocking BMP binding to the receptor [103]. Hatsell et al. [101] used a specific conditional-on knock-in ACVR1^R206H^ mouse and showed enhanced heterotopic ossification induced by activin A which could be inhibited by the use of activin A neutralizing antibodies [101, 103]. Accordingly, implantation of ACVR1(R206H)-transfected myoblastic C2C12 cells in nude mice caused heterotopic ossification in muscle tissue [104]. Together, these data indicate not only an important crosstalk between myogenesis and osteogenesis but also point to an important role for activin A in bone remodelling. However, the increased formation of osteoclasts in the muscle tissue after transplantation of transfected myoblastic C2C12 cells was not dependent on activin A but on TGF-β1 [104].

Nevertheless, the induction of activin A in many cell types by pro-inflammatory cytokines in response to injury and inflammation makes a crosstalk also between inflammation and osteogenesis most likely.

## The role of activin A in inflammatory bone destruction

Activin A is expressed in FLS of the lining and sublining layers and by macrophages in the active RA synovium. Expression of activin A by FLS is stimulated by pro-inflammatory cytokines such as TNF-α, IL-1β and TGF- β; IL-1β appears to be the strongest inducer of activin A secretion by FLS [90]. Moreover, activin A is expressed in α-smooth muscle actin (αSMA) high-expressing FLS from high-inflammatory synovial tissues of RA patients, further confirming an important role in the pathogenesis of RA [105].

Additionally, higher levels of activin A in the synovial fluid of RA patients compared to systemic lupus erythematosus, OA and control patients could be observed [106]. Interestingly, high levels of activin A present in the synovial fluid of RA patients were generated by granulocyte-macrophage colony stimulating factor (GM-CSF)-dependent differentiated pro-inflammatory macrophages (M1) [91, 107]. Thus, activin A from the synovial fluid of RA patients contributes to the polarisation towards pro-inflammatory GM-CSF-mediated M1 macrophages, inhibits lipopolysaccharide-mediated IL-10 secretion by M2 macrophages and decreases the expression of anti-inflammatory M2 (M-CSF)-dependent markers in monocytes and macrophages [91, 107]. This supports the notion that activation and polarization of synovial fluid macrophages by activin A are important processes for pathogenic progression and severity of RA in the affected joints [91].

In fact, it has been shown that activin A can act as a pro- or anti-inflammatory mediator, which is dependent on its concentration at the site of inflammation. High activin A levels promote anti-inflammatory processes, whereas low levels exert most likely pro-inflammatory effects, which could be associated with the activation of pro- or anti-inflammatory macrophages (reviewed by Dong and He [108]).

In this regard, enhanced production of activin A could be observed after stimulation of monocytes derived from human peripheral blood with synovial fluid of RA patients and macrophages from the synovial fluid of RA patients showed a higher expression of p-R-Smad2 in comparison to pro-inflammatory or anti-inflammatory macrophages derived from peripheral blood mononuclear cells [91].

Besides a direct role on macrophages and osteoclasts, activin A appears to have an additionally indirect role in bone destruction in RA. Of interest, activin A has the capability to induce the proliferation of synovial fibroblasts, suggesting a role for it in the formation of an invasive pannus that destroys articular cartilage and bone in RA joints [90]. Indeed, many studies revealed a higher expression of RANKL in synovial tissues and synovial fibroblasts from RA compared to OA patients and exclusively co-cultures with synovial fibroblasts from patients with RA promote osteoclast development in vitro, indicating that upregulation of RANKL in activated synovial fibroblasts may facilitate the differentiation of osteoclasts [109, 110]. Thus, enhanced formation of pannus tissue by activin A, associated with increased levels of RANKL, may be responsible for increased bone destruction in RA.

## Future perspectives on the treatment of RA

The present data suggest that the inhibition of certain members of the TGF-β superfamily and their downstream, Smad-dependent signalling pathways may be of benefit for RA and constitute novel, so far incompletely explored treatment options. This notion is based on a number of observations as outlined in this review. First, the data indicate that particularly the inhibition of Smad-2 activation may exert effects that are distinct from other signalling pathways and thus provide additional benefit over existing and recently explored targeting options such as MAPK or JAK/STAT inhibition. Second, it appears that, in addition to their classic roles, e.g. of myostatin on muscle biology, Smads, and again specifically Smad-2, are involved in the shuttling and activation of other transcription factors (e.g. c-fos, NFATc1) that control one key aspect of RA pathogenesis, namely bone destruction. Inhibition of these pathways may, therefore, combine strong anti-resorptive effects with benefits in other, more systemic disease manifestations, such as muscle weakness or inflammation. Certainly, a number of important questions remain to be answered. These relate both to the exact mechanisms by which the activation of Smads and their upstream activating receptors regulate and link the different disease mechanism of RA and also to required dosages and potential unwanted effects. Nonetheless, the current evidence not only warrants further exploration of the underlying signalling pathways and their relevance for RA but also the initiation of furher (pre-)clinical steps towards the use of existing compounds such as myostatin inhibitors.

## Conclusions

RA is a chronic inflammatory disease that is characterised by progressive destruction of the joints, mainly caused by osteoclastic bone resorption. Osteoclasts differentiate from hematopoietic precursors or tissue-resident macrophages by RANKL, a key factor of osteclast differentiation, expressed by synovial fibroblasts and activated T cells and regulated by pro-inflammatory cytokines which are highly expressed in the inflamed pannus tissue as well. Although RANKL is essential for osteoclast formation, other factors, which additionally are abundant in the synovial membrane or bone of patients with RA, are able to potently stimulate osteoclast development. In this regard, TGF-β1, myostatin and activin A, all members of the TGF-β family, enhance RANKL-induced osteoclast formation, suggesting an important role of Smad signalling in the regulation of osteoclast differentiation and inflammatory bone loss. The identification of additional players in inflammatory bone resorption may contribute significantly to the discovery of new and better drugs for the treatment of joint destruction in arthritis.

## Abbreviations

ALK: Activin receptor-like kinase
AP1: c-fos/c-jun hetero- and homodimeric transcription factors
BMM: Bone marrow-derived macrophage
BMP: Bone morphogenetic protein
CIA: Collagen-induced arthritis
Co-Smad: Common-mediator Smad
FLS: Synovial-like fibroblast
FOP: Fibrodysplasia ossificans progressive
GDF: Growth and differentiation factor
GM-CSF: Granulocyte-macrophage colony stimulating factor
hTNFtg: human tumor necrosis factor α transgenic mice
IL: Interleukin
I-Smad: Inhibitory Smad
JNK1: c-Jun N-terminal kinase 1
MAPK: Mitogen-activated protein kinase
M-CSF: Macrophage colony stimulating factor
MMP: Matrix metalloproteinase
NF-κB: nuclear factor kappa-light-chain-enhancer of activated B-cells
NFATc1: Nuclear factor of activated T-cells cytoplasmic 1
OA: Osteoarthritis
OPG: Osteoprotegerin
RA: Rheumatoid arthritis
RANK: Receptor activator of nuclear factor κB
RANKL: Receptor activator of nuclear factor κB ligand
R-Smad: Receptor-regulated Smad
TGF: Transforming growth factor
TNF: Tumour necrosis factor
TRAF6: TNF receptor-associated factor 6
TRAP: Tartrate-resistant acid phosphatase.
